# Where Do We Stand in the Management of Rheumatoid Arthritis Ahead of EULAR/ACR 2025?

**DOI:** 10.3390/clinpract15060103

**Published:** 2025-05-28

**Authors:** Adriana Liliana Vlad, Corina Popazu, Alina-Maria Lescai, Doina Carina Voinescu, Alexia Anastasia Ștefania Baltă

**Affiliations:** 1 Faculty of Medicine and Pharmacy, “Dunărea de Jos” University of Galați, 800008 Galați, Romania; adriana.vlad.mg3.4@gmail.com (A.L.V.); corinapopazu@yahoo.com (C.P.); carinavoinescu@gmail.com (D.C.V.); alexiabalta@yahoo.ro (A.A.Ș.B.); 2 “St. Apostle Andrei” Clinical Emergency County Hospital, 800578 Galați, Romania

**Keywords:** rheumatoid arthritis, interdisciplinary approach, DMARDs, corticosteroids, NSAIDs, inflammatory monitoring, imaging, psychosocial support, physical rehabilitation, personalised management

## Abstract

**Background**: Rheumatoid arthritis (RA) is a chronic autoimmune disease characterised by systemic inflammation and the progressive damage of joints, significantly impacting patients’ quality of life. Managing this condition requires a complex approach that integrates pharmacological and non-pharmacological therapies, alongside psychosocial support and patient education. Aim: This study aims to highlight the importance of an interdisciplinary approach in the treatment of rheumatoid arthritis, focusing on the role of pharmacological therapies, monitoring treatment response, and the involvement of a multidisciplinary team in the effective management of the disease. **Methods**: The analysis was based on a review of the specialised literature concerning the role of disease-modifying antirheumatic drugs (DMARDs, both conventional and biological), the use of inflammatory markers (CRP and ESR), advanced imaging techniques, and the contribution of various medical specialities to the holistic management of rheumatoid arthritis. A total of 595,900 records were identified, of which 53 studies were ultimately included in the detailed analysis. Relevant studies from fields such as rheumatology, nutrition, psychology, and physical therapy were included. **Results**: The findings underline that DMARDs, in combination with other pharmacological therapies, remain essential for slowing disease progression. Monitoring treatment response through inflammatory markers and imaging techniques allows for the adjustment of therapeutic strategies and the prevention of complications. An interdisciplinary approach, involving the rheumatologist, general practitioner, physiotherapist, nutritionist, and psychologist, provides significant benefits, such as reducing inflammation, improving joint function, and offering psychosocial support. **Conclusions**: The effective management of rheumatoid arthritis requires a personalised interdisciplinary approach. Integrating various specialities, along with patient education and psychosocial support, contributes to better disease management, the prevention of disabilities, and improved quality of life. This review is not registered.

## 1. Introduction

Rheumatoid arthritis (RA) is a chronic autoimmune disease characterised by persistent inflammation predominantly affecting synovial joints [1]. It leads to pain, swelling, and joint stiffness, often symmetrically, with the wrists, knees, and feet being the most commonly involved joints [2]. The inflammatory process is not confined to the joints but may also involve other organs and systems, such as the skin, lungs, heart, and blood vessels [3].

Rheumatoid arthritis is among the most common autoimmune inflammatory diseases, affecting approximately 0.5–1% of the general population worldwide [4]. The increasing prevalence of rheumatoid arthritis, particularly in developed countries, highlights the growing healthcare burden, demanding not only pharmacological interventions but also an interdisciplinary management approach to ensure holistic care for patients. Its prevalence varies by region, being higher in developed countries and communities with greater access to diagnosis and treatment but lower in some parts of Africa and Asia, where healthcare access is limited, or underdiagnosis remains an issue [5,6].

The condition is two to three times more common in women than men, with this disparity being most pronounced during the reproductive years, suggesting an important role for hormonal factors [7]. The risk of developing rheumatoid arthritis increases with age; although, the disease can manifest at any stage of life [8].

Factors such as smoking, occupational exposure to certain chemicals, and a family history of autoimmune diseases contribute to the variability in prevalence across different populations [9,10]. Furthermore, rheumatoid arthritis’ systemic nature not only increases mortality risks due to cardiovascular complications but also places a significant emotional and economic burden on patients, underscoring the need for multidisciplinary collaboration to address these broader impacts [11]. Recent epidemiological studies underscore the importance of early diagnosis and access to modern treatments, which can significantly impact the progression and prognosis of the disease [12,13].

An interdisciplinary approach is especially valuable in rheumatoid arthritis management, as it brings together experts from multiple disciplines to address the physical, psychological, and social dimensions of the disease. This collaborative method fosters communication and coordination among professionals, ensuring that strategies are tailored to the specific needs of each patient. Unlike traditional, siloed approaches where specialists work independently, interdisciplinary management aligns diverse interventions into a cohesive framework.

For example, while a rheumatologist may focus on controlling inflammation through DMARDs or corticosteroids, a physiotherapist addresses joint mobility, and a psychologist helps manage the emotional toll of living with a chronic condition. Nutritionists also play a critical role by designing dietary strategies that complement pharmacological treatments and reduce systemic inflammation. Together, these professionals provide patient-centred care that acknowledges the multifactorial nature of rheumatoid arthritis.

This integration of expertise is particularly effective in rheumatoid arthritis, because it ensures that not only are the symptoms of the disease managed but the broader impacts—such as reduced quality of life, emotional distress, and economic challenges—are also addressed. Interdisciplinary management enhances the efficacy of pharmacological treatments by incorporating non-pharmacological strategies, such as physiotherapy, psychosocial support, and nutritional counselling.

The paper offers a novel contribution by integrating the interdisciplinary approach as a central element in the treatment and management of rheumatoid arthritis. While pharmacological therapy remains a cornerstone of rheumatoid arthritis management, this review provides a holistic perspective that includes several innovative aspects. It highlights the integration of pharmacological interventions with non-pharmacological strategies within an interdisciplinary context. The paper explores how drug therapies, such as NSAIDs, corticosteroids, and DMARDs, can be combined with non-pharmacological interventions, including physiotherapy, psychosocial support, and nutritional strategies, emphasising the benefits of a coordinated approach. Unlike previous studies that separately analyse treatments or rheumatoid arthritis comorbidities, this review details the specific role of each member of the interdisciplinary team, including rheumatologists, physiotherapists, nutritionists, and psychologists. It demonstrates how collaboration among specialists improves clinical outcomes and enhances patients’ quality of life. A patient-centred perspective is also introduced in rheumatoid arthritis management. Emphasis is placed on patient education, psychological support, and self-management strategies, which are often overlooked in studies focused solely on pharmacotherapy. The review advocates for the active involvement of patients in decision making as part of the interdisciplinary team.

The paper identifies gaps and opportunities for future research, highlighting the challenges of implementing interdisciplinary care, such as limited access to multidisciplinary teams and the absence of standardised protocols. It provides concrete directions for further studies to optimise these strategies. By synthesising recent data from 2020 to 2024, this review distinguishes itself from earlier studies by focusing on contemporary advancements. These include the use of novel biomarkers, such as calprotectin, and advanced imaging techniques, including ultrasound and MRI, for personalised monitoring of disease progression.

This review is grounded in a comprehensive analysis of 104 references, reflecting a wide array of high-quality sources that span recent advancements in the interdisciplinary management of rheumatoid arthritis. These references were meticulously selected to ensure both breadth and depth, incorporating systematic reviews, meta-analyses, clinical trials, and observational studies published in reputable journals.

The extensive bibliography includes studies on pharmacological interventions, such as NSAIDs, corticosteroids, and DMARDs, as well as non-pharmacological strategies, like physiotherapy, nutritional approaches, and psychosocial support. Additionally, it encompasses cutting-edge research on biomarkers, like calprotectin, advanced imaging techniques including ultrasound and MRI, and emerging trends in personalised care.

By integrating findings from diverse disciplines—rheumatology, psychology, nutrition, physiotherapy, and beyond—this review demonstrates a holistic approach to rheumatoid arthritis management. The references also provide critical insights into the systemic nature of rheumatoid arthritis, highlighting comorbidities, socioeconomic burdens, and the necessity of patient-centred care within an interdisciplinary framework.

The inclusion of 104 references underscores the rigour and comprehensiveness of this review, offering a well-rounded synthesis of contemporary knowledge and serving as a reliable foundation for identifying gaps, exploring implications, and proposing directions for future research.

### 1.1. Pathophysiology and Causes of Rheumatoid Arthritis

Rheumatoid arthritis is a complex condition, with its pathophysiological mechanism driven by an autoimmune process [14]. The immune system mistakenly identifies self-structures, particularly the synovial membrane lining the joints, as foreign [15]. This triggers a chronic inflammatory reaction in which T and B lymphocytes, as well as macrophages, play central roles [16]. These immune cells stimulate the production of inflammatory cytokines, such as tumour necrosis factor-alpha (TNF-α) and interleukins, which amplify inflammation and contribute to progressive tissue damage [17].

To better contextualise the impact of chronic inflammation on joint destruction, an emphasis on the role of pannus formation as a dynamic contributor to joint erosion and dysfunction is critical. The interplay between pannus tissue and immune cell activity creates a self-perpetuating cycle of inflammation that necessitates targeted therapeutic intervention.

Genetic factors significantly influence the risk of developing rheumatoid arthritis. Specific alleles, such as those in the major histocompatibility complex (HLA-DR4 and HLA-DR1), are associated with increased susceptibility [18]. However, the onset of the disease typically requires interaction with environmental factors. Smoking, for instance, increases the risk and severity of the disease by promoting autoimmune changes [19]. Other environmental elements, such as bacterial or viral infections, may act as triggers in genetically predisposed individuals [20].

Chronic inflammation plays a decisive role in the joint destruction characteristic of rheumatoid arthritis [21]. Persistent synovitis, marked by excessive proliferation of synovial cells and tissue infiltration by immune cells, leads to the formation of a pathological tissue called pannus [22]. Therapeutic strategies targeting pannus formation and immune dysregulation have shown promise in recent research, particularly through biologic DMARDs that inhibit specific cytokines, such as TNF-α and interleukins.

This pannus invades the cartilage and subchondral bone, causing erosions, loss of joint structure, and irreversible deformities [23]. Without appropriate treatment, chronic inflammation can also affect other organs, exacerbating the disease burden on the patient [24]. Given this complexity, interdisciplinary approaches that integrate rheumatology, immunology, and cardiology are essential to address both joint-specific and systemic manifestations of rheumatoid arthritis.

### 1.2. Clinical Signs and Symptoms

Rheumatoid arthritis typically begins with subtle early manifestations, including fatigue, prolonged morning joint stiffness, and diffuse joint pain [25]. These initial symptoms are often symmetrical and predominantly affect the small joints of the hands, wrists, and feet [26]. As the disease progresses, symptoms become more pronounced, with visible joint swelling, restricted movement, and characteristic deformities [27]. In advanced stages, structural damage leads to significant functional disability, and affected joints may become completely immobilised [28].

One notable gap in current diagnostic protocols is the underestimation of subclinical inflammation, which may be present even in patients with minimal or no visible symptoms. Advanced imaging techniques, particularly MRI and ultrasound, can play a crucial role in detecting these early changes and initiating timely interventions to prevent irreversible damage.

In addition to joint involvement, rheumatoid arthritis can cause extra-articular manifestations, reflecting its systemic nature. These include rheumatoid nodules, which appear under the skin in pressure areas, such as the elbows, and organ involvement [29]. Rheumatoid vasculitis, a severe extra-articular manifestation, remains underdiagnosed and requires a high degree of clinical suspicion for timely identification and management.

The lungs may be affected, leading to pleurisy or pulmonary fibrosis, while chronic inflammation may impact the heart, causing pericarditis or increasing the risk of cardiovascular disease [30]. Severe dryness and inflammation can occur in the eyes, and blood vessels may be affected by rheumatoid vasculitis [31].

Rheumatoid arthritis is frequently associated with comorbidities that complicate disease management. The most common include osteoporosis, accelerated by chronic inflammation and corticosteroid use, and cardiovascular diseases driven by systemic inflammatory effects [32]. Other associated conditions include diabetes mellitus, metabolic syndrome, and depression, all of which negatively impact patients’ quality of life [33]. Recognising and addressing these comorbidities is essential for comprehensive disease management. Addressing these comorbidities necessitates close collaboration between rheumatologists, cardiologists, and endocrinologists to ensure comprehensive care.

### 1.3. Diagnosis of Rheumatoid Arthritis

Symptoms persisting for more than six weeks are also an important diagnostic criterion.

Updated classification criteria, such as the ACR/EULAR 2010 criteria, provide a more nuanced framework for identifying early-stage rheumatoid arthritis, incorporating serological markers and imaging findings for improved sensitivity.

**Figure 1:**
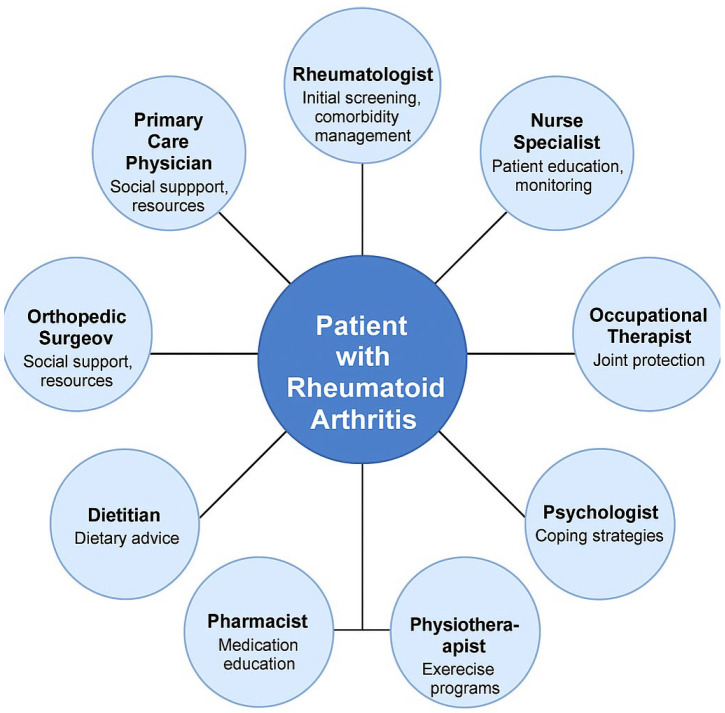
Interdisciplinarity in treatment.

Imaging plays a vital role in diagnosis [Figure 1]. X-rays can reveal bone erosions and joint space narrowing in advanced stages, while ultrasound or magnetic resonance imaging (MRI) can detect inflammation and structural changes even in the early stages [34]. While these imaging modalities have advanced significantly, accessibility and cost barriers remain key challenges in their widespread adoption, particularly in low-resource settings. Addressing these gaps requires policy-level interventions to enhance healthcare equity.

Laboratory tests are essential for confirming the diagnosis and assessing disease severity. Rheumatoid factor (RF), an autoantibody found in most patients, is an important marker, though not exclusive to rheumatoid arthritis [35]. More specific are anti-cyclic citrullinated peptide (anti-CCP) antibodies, which have high sensitivity and specificity, particularly in early-stage disease [36]. Inflammatory markers, such as C-reactive protein (CRP) and erythrocyte sedimentation rate, are used to monitor disease activity but are not specific to rheumatoid arthritis [37]. In addition, novel biomarkers, such as calprotectin and haematological ratios (e.g., neutrophil-to-lymphocyte ratio), are gaining attention as complementary tools in the diagnostic arsenal, warranting further exploration.

The integrated interpretation of clinical, imaging, and laboratory data is crucial for an accurate diagnosis and for initiating appropriate treatment.

**Figure 2:**
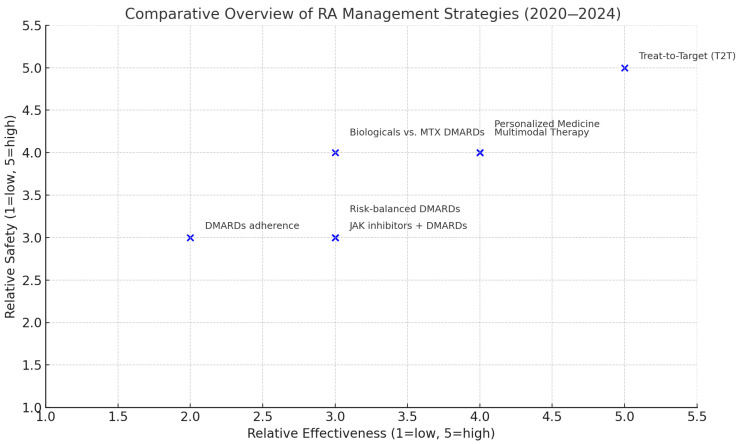
Management strategies.

Below is a meta-analytical comparative overview of rheumatoid arthritis (RA) management strategies (2020–2024), see Figure 2.

This figure presents a meta-analytical synthesis of the relative effectiveness and safety of key pharmacologic and management strategies used in rheumatoid arthritis, based on data extracted from recent systematic reviews and meta-analyses conducted between 2020 and 2024. The purpose of this meta-analysis is to consolidate heterogeneous evidence into a single comparative framework, offering clinicians and researchers an accessible visual tool to guide therapeutic decision making. By integrating outcomes from multiple high-quality studies, meta-analyses enhance statistical power and allow for more precise estimates of intervention effects across diverse patient populations.

The figure displays relative scores for both efficacy and safety, with values ranging from 1 (low) to 5 (high). Methotrexate (MTX), both oral and subcutaneous, remains the cornerstone of conventional synthetic DMARD therapy due to its proven balance of effectiveness and tolerability. Emerging agents, such as ozoralizumab and iguratimod, administered alone or in combination with MTX, have shown improved clinical response with favourable safety profiles. Biologic DMARDs (bDMARDs) and their biosimilars demonstrate equivalent outcomes across various RA cohorts. The combination of JAK inhibitors with MTX yields similar efficacy and safety to tumour necrosis factor inhibitors (TNFi). The treat-to-target (T2T) approach shows the highest combined ratings for efficacy and safety, highlighting the importance of proactive and structured disease monitoring. Persistent challenges remain regarding medication adherence, particularly with conventional DMARD therapies. This meta-analytical overview reinforces the need for personalized treatment strategies and interdisciplinary care pathways to optimize long-term clinical outcomes in patients with rheumatoid arthritis.

## 2. Materials and Methods

The PRISMA (Preferred Reporting Items for Systematic Reviews and Meta-Analyses) model is a set of guidelines designed to enhance the quality of reporting in systematic reviews and meta-analyses. It helps researchers transparently describe how such studies are planned, conducted, and reported. PRISMA is recognised as the standard of excellence for reporting synthesis research [38,39]. This review is not registered.

**Figure 3:**
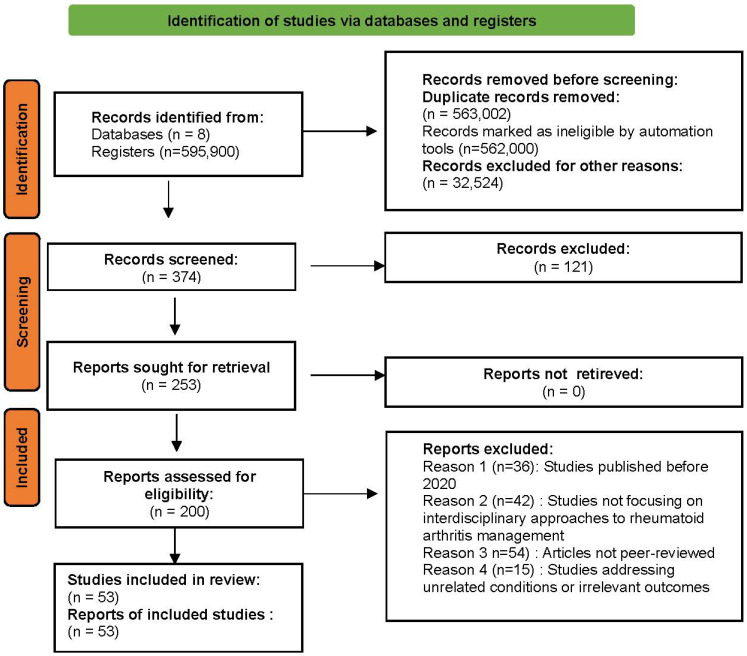
PRISMA flow diagram of articles related to interdisciplinary approaches in rheumatoid arthritis management.

To ensure transparency and reproducibility, the PRISMA framework was rigorously applied in this review. The inclusion of a detailed PRISMA flow diagram (Figure 3) enables readers to clearly follow the selection process and understand the criteria for the inclusion and exclusion of studies. However, additional attention was given to explaining discrepancies between the first and second review phases to address feedback regarding clarity in methodology.

The type of review conducted in this study can be defined as a systematic narrative review. It combines elements of a systematic review, which involves a rigorous and well-documented methodology for identifying, selecting, and analysing relevant studies, with a narrative approach that synthesises and integrates findings from a diverse range of sources to provide a coherent overview of a complex subject.

The findings from the included studies were synthesized and presented narratively, focusing on explanations and discussions that emphasize the complexity of interdisciplinary management in rheumatoid arthritis. Beyond reporting concrete data, the synthesis also explored clinical implications, knowledge gaps, and future research opportunities.

This systematic narrative review was selected due to the complexity of the topic, which encompasses a broad spectrum of interventions and requires insights from multiple medical and complementary disciplines. An explanatory framework was essential for synthesizing data and identifying gaps and directions for future research.

This review focused on the following three primary dimensions of the interdisciplinary approach:

- Pharmacological interventions, including NSAIDs, corticosteroids, and DMARDs, within a collaborative framework.
- Non-pharmacological strategies, such as physiotherapy, dietary interventions, and psychosocial support.
- The role of a multidisciplinary team in enhancing patient outcomes, including the integration of rheumatologists, general practitioners, psychologists, and rehabilitation specialists.

A key research question was as follows: How does an interdisciplinary approach influence the effectiveness of treatment and overall management of rheumatoid arthritis, particularly in terms of controlling inflammation, preventing disease progression, and improving patients’ quality of life?

The exploration of interdisciplinary management in rheumatoid arthritis focused on integrating various approaches and disciplines to enhance patient care. Pharmacological interventions, including NSAIDs, corticosteroids, and DMARDs, were examined within collaborative frameworks to ensure their effective application alongside complementary strategies. Non-pharmacological methods, such as physiotherapy, dietary modifications, and psychosocial support, were assessed for their role in improving quality of life and managing symptoms. Particular attention was given to the contributions of multidisciplinary teams, highlighting how the coordination between rheumatologists, general practitioners, psychologists, nutritionists, and rehabilitation specialists can optimise outcomes for patients with rheumatoid arthritis. Also, this exploration addressed gaps in current knowledge, identified barriers to interdisciplinary collaboration, and proposed opportunities for future research to refine and expand these approaches.

### 2.1. Approach to Identifying Relevant Articles

To address the research question, a systematic search was conducted across the following eight databases: MDPI, Wiley Online Library, De Gruyter, Taylor & Francis, Springer, Frontiers, Nature, and Elsevier.

The search strategy followed PRISMA guidelines, employing a robust combination of Boolean operators (AND, OR) to refine and optimise results. This ensured comprehensive coverage of interdisciplinary approaches and rheumatoid arthritis management. 

Key search terms included the following: “rheumatoid arthritis” (139,000 results), “interdisciplinary management” (380,000 results), “DMARDs” (16,900 results), “NSAIDs” (24,000 results), and “corticosteroids” (36,000 results).

These terms were combined to create structured search strings, such as “(rheumatoid arthritis) AND (interdisciplinary management) AND (NSAIDs OR DMARDs)” to maximise relevance.

Filtering was performed using the following criteria: publication period of 2020–2024 and availability of full texts in English.

Each database was queried using a tailored approach to account for differences in search functionalities. In addition, snowballing techniques were applied, where references cited in the included articles were manually reviewed to identify any overlooked studies.

### 2.2. Eligibility Criteria for Study Selection

#### 2.2.1. Inclusion Criteria

The selected articles had to address rheumatoid arthritis management from an interdisciplinary perspective. Publications were required to include information on the following:

- Pharmacological treatments (NSAIDs, corticosteroids, DMARDs) within a multidisciplinary care framework.
- Monitoring tools, such as inflammatory markers (e.g., CRP, ESR) and imaging techniques (e.g., ultrasound, MRI).
- Non-pharmacological interventions, including physiotherapy, dietary strategies, and psychosocial support.

The review included original studies, meta-analyses, systematic reviews, and research articles published in indexed journals, ensuring high-quality sources.

#### 2.2.2. Exclusion Criteria

The following were excluded:

- Studies not adhering to PRISMA guidelines, as compliance is critical for methodological rigor.
- Case studies without generalisable findings or lacking a clear interdisciplinary focus.

#### 2.2.3. Selection Process

All identified articles were evaluated based on the inclusion and exclusion criteria to ensure the relevance and quality of the final sample. The PRISMA framework guided the entire selection process, with the final pool of studies reflecting diverse aspects of rheumatoid arthritis management. To address feedback, we provide additional clarification regarding the two review phases, as follows:

In the first phase, studies were screened for general relevance to interdisciplinary rheumatoid arthritis management based on abstracts and titles.

In the second phase, a full-text analysis was conducted, focusing on studies that explicitly reported interdisciplinary approaches, such as combined pharmacological and non-pharmacological strategies or multidisciplinary care models.

The final set of studies underwent a detailed quality assessment to confirm their compliance with PRISMA standards and relevance to the research question.

These measures demonstrate that the selection process was both comprehensive and precise, minimising the risk of omitting relevant studies.

Although the described process adheres to PRISMA standards, certain types of bias may influence the selection of articles, such as the exclusion of grey literature, which limits access to less visible research, the restriction to articles in English, which may omit valuable studies published in other languages, the exclusive use of eight major databases, which could overlook other relevant sources, and the limitation of the publication period to 2020–2024, potentially disregarding earlier studies with useful perspectives.

### 2.3. Risk of Bias and Evidence Quality Assessment

To enhance the methodological transparency and reliability of this systematic narrative review, an overall assessment of the risk of bias and evidence quality was conducted during the full-text screening phase. The evaluation was guided by the principles of the Cochrane risk of bias tool for randomized controlled trials and adapted criteria for observational studies, including the clarity of methodology, consistency of data reporting, and adherence to predefined inclusion criteria. Due to the narrative design and broad scope of the review, a detailed, study-by-study risk of bias matrix was not applied. Instead, an overall appraisal of the quality of evidence was provided across the included studies. The main limitations of the selected studies and the potential sources of bias have been acknowledged and discussed in the Results and Discussion sections. This methodological approach was considered appropriate to balance the comprehensive nature of the synthesis with the practical constraints of a narrative systematic review.

## 3. Results

### 3.1. Overview of Selected Studies

The PRISMA flow diagram presented in Figure 1 illustrates the selection process for articles related to rheumatoid arthritis management. A total of eight databases were searched, generating 595,900 results.

A total of eight databases were searched, generating 595,900 results. After removing duplicate records (563,002) and those excluded for other reasons (562,628) (e.g., lack of relevance, incomplete data), 374 articles remained for screening. During the screening phase, 121 records were excluded based on the predefined criteria, primarily due to their limited focus on interdisciplinary management or insufficient adherence to PRISMA standards.

In the first review phase, 253 reports were assessed for eligibility. These studies were evaluated for general relevance to rheumatoid arthritis management, resulting in 200 studies being included for further analysis.

In the second phase, a more focused evaluation was conducted, applying stricter inclusion criteria regarding interdisciplinary care. Studies that exclusively covered monotherapy or lacked a multidisciplinary approach were excluded.

Studies spanned a variety of interdisciplinary approaches in rheumatoid arthritis management and were published between 2020 and 2024.

This methodology enabled a comprehensive exploration of the specialised literature, synthesising relevant information on modern and interdisciplinary approaches to the management of rheumatoid arthritis.

The studies presented in the Results section are those that strictly adhered to the inclusion criteria established in the methodology, providing objective and focused evidence to address the research question. These studies were systematically selected and synthesised to evaluate the direct impact of interdisciplinary approaches on rheumatoid arthritis management, offering quantifiable data and concrete outcomes relevant to the scope of the review.

### 3.2. The Role of NSAIDs in the Management of Rheumatoid Arthritis

Pharmacological therapy is a cornerstone in the management of rheumatoid arthritis, with non-steroidal anti-inflammatory drugs (NSAIDs) playing a key role in controlling inflammatory symptoms and pain. Table 1 summarises the uses, benefits, limitations, and clinical considerations of NSAIDs in the treatment of this condition.

From Table 1, NSAIDs remain essential for rapid relief of inflammatory symptoms, particularly in acute disease phases, but are not suitable as standalone therapy due to their inability to modify disease progression.

Integrated therapeutic plans, combining NSAIDs with DMARDs or biologics, were shown to improve long-term outcomes, while minimising NSAID-associated risks.

### 3.3. The Role of Corticosteroids in the Management of Rheumatoid Arthritis

Corticosteroids are a crucial therapeutic option in the management of rheumatoid arthritis, offering the rapid control of inflammation and relief of acute symptoms. Table 2 provides a detailed overview of their mechanism of action, benefits, clinical uses, risks, and associated limitations.

As shown in Table 2, short-term corticosteroid use can significantly improve quality of life by rapidly controlling inflammation, but long-term strategies require close monitoring to mitigate risks.

The integration of corticosteroid therapy into a broader interdisciplinary framework (e.g., combining with physiotherapy and dietary support) was shown to enhance overall outcomes.

### 3.4. Comparison Between Conventional and Biologic DMARDs in the Treatment of Rheumatoid Arthritis

To better understand the role of disease-modifying antirheumatic drugs (DMARDs) in the management of rheumatoid arthritis, it is helpful to examine the differences between the two main categories—conventional and biologic DMARDs. Table 3 provides a detailed comparison of the characteristics, efficacy, risks, and limitations of each therapy type, highlighting the benefits and challenges associated with their use.

Comparing conventional and biologic DMARDs highlights the distinct roles each category plays in the treatment of rheumatoid arthritis (Table 3). Conventional DMARDs remain the foundation of treatment, offering accessibility and effectiveness, while biologics provide a high-performance option for more severe or refractory cases. Choosing the appropriate therapy requires a personalised approach, careful monitoring, and often, integration with other therapeutic strategies to achieve optimal disease management.

### 3.5. Monitoring the Response to Rheumatoid Arthritis Treatment

Monitoring the response to rheumatoid arthritis treatment is essential to assess the effectiveness of therapeutic interventions and to adapt strategies based on disease progression.

Abdelhafiz et al. (2023) emphasise the pivotal role of inflammatory markers, such as C-reactive protein (CRP) and erythrocyte sedimentation rate in tracking inflammation and disease activity in rheumatoid arthritis [77]. Pope et al. (2021) argue that CRP is a sensitive marker of acute inflammation, reflecting both disease activity and short-term treatment response [78]. Conversely, Targońska-Stępniak et al. (2020) highlight that, while the erythrocyte sedimentation rate is less specific, it provides valuable insights into systemic inflammation and is often used alongside CRP for a more comprehensive evaluation [79]. Ullah et al. (2024) suggest that elevated levels of these markers indicate heightened disease activity, warranting therapy adjustments, while their reduction signals adequate control of inflammation [80].

Conforti et al. (2021) advocate for the indispensable role of imaging techniques in monitoring rheumatoid arthritis progression and detecting structural changes, which may occur even when inflammation appears to be under control [81]. Imtiaz et al. (2022) note that conventional radiographs are useful for observing bone erosions and joint space narrowing in advanced stages [82]. However, Elangovan et al. (2020) point out that ultrasound provides more detailed information on active inflammation and early tissue changes, being an accessible and non-invasive tool [34]. Weaver et al. (2022) assert that MRI is superior for detecting early inflammation and subclinical lesions in cartilage and bone [83].

Integrating inflammatory markers and imaging techniques into rheumatoid arthritis monitoring enables a personalised treatment approach, reducing the risk of disease progression and long-term disability. This combined strategy allows the medical team to intervene promptly when treatment response is suboptimal, ensuring optimal control of rheumatoid arthritis.

### 3.6. The Interdisciplinary Approach in Treatment and Management

An interdisciplinary approach is crucial in the treatment and management of rheumatoid arthritis, given the complexity of the disease and its impact on multiple aspects of patients’ lives. A multidisciplinary team ensures holistic, coordinated, and effective management.

Interdisciplinary management is an approach to problem solving and decision making that involves the collaboration of professionals from diverse disciplines, integrating their knowledge, skills, and perspectives to address complex issues comprehensively. It emphasises coordinated efforts, mutual respect, and shared goals to achieve outcomes that are more effective and holistic than those achieved through single-discipline approaches.

The rheumatologist is the central figure, responsible for diagnosis, initiating and adjusting pharmacological treatments, monitoring disease progression, and coordinating with other team members. Gukova et al. (2022) emphasise that the rheumatologist leads the team, with primary responsibility for diagnosis, initiating and adjusting treatments, and monitoring disease progression, serving as the cornerstone of rheumatoid arthritis management [84].

General practitioners and specialists in other fields (cardiologists, pulmonologists, endocrinologists) are involved in managing common comorbidities, such as cardiovascular disease, osteoporosis, and diabetes, which often complicate rheumatoid arthritis progression. Bech et al. (2020) highlight the role of the general practitioner, who provides ongoing patient monitoring, manages comorbidities, and implements preventive measures to improve patients’ quality of life [85].

The physiotherapist contributes by developing personalised exercise programmes aimed at maintaining joint mobility, reducing pain, and preventing muscle atrophy. These programmes are tailored to the stage of the disease and the patient’s specific needs. Mohapatra et al. (2023) underline the importance of physiotherapists and kinesiotherapists, who support mobility maintenance and improvement through personalised exercises that reduce pain and enhance the functionality of affected joints [86].

The nutritionist plays a vital role in creating anti-inflammatory dietary plans, which include foods rich in omega-3 fatty acids and antioxidants. Maintaining an optimal body weight reduces stress on the joints and improves overall health. Nikiphorou and Philippou (2023) stress the role of the nutritionist in crafting tailored dietary plans focused on anti-inflammatory diets, which help reduce inflammation and maintain an optimal body weight, both of which influence disease progression [87].

The psychologist is crucial in supporting patients in managing the emotional impact of a chronic illness. Psychological interventions, such as cognitive behavioural therapy, can alleviate anxiety, depression, and stress associated with rheumatoid arthritis. Nagy et al. (2023) highlight the critical role of the psychologist in providing psychosocial support, helping patients manage the emotional stress of chronic illness, and developing effective coping strategies for daily challenges [88]. This aspect is consistent with the conclusions of Anghele, M. et al. (2023), who emphasize the importance of psychological intervention to prevent the progression of mental pathologies and to improve the general emotional state of polytraumatized patients [89].

From our practical experience, social workers and occupational therapists provide support for social and professional integration, helping patients adapt their daily activities and use assistive devices to maintain autonomy. The interdisciplinary management of rheumatoid arthritis also involves social workers and occupational therapists, who provide essential support for patients’ integration into social and professional environments. Social workers help patients navigate social services and secure resources, while occupational therapists assist with adapting daily activities and incorporating assistive devices to maintain independence.

Also, geriatricians play an emerging role, particularly given the increasing prevalence of rheumatoid arthritis in older adults. Their expertise in managing age-related comorbidities, polypharmacy, and frailty adds an essential layer to holistic care. Similarly, palliative care specialists can support patients in advanced disease stages, focusing on pain management and improving quality of life when curative strategies are limited.

Di Ludovico et al. (2023) point to the importance of physical rehabilitation, which includes exercises tailored to the patient’s needs to maintain mobility, reduce stiffness, and prevent muscle atrophy [90]. Gavin et al. (2024) emphasise the value of occupational therapy, which helps patients adapt daily activities and utilise assistive devices to enhance autonomy and improve quality of life [91]. The benefits of this interdisciplinary collaboration extend beyond the clinical realm. Patients who benefit from a multidisciplinary approach often experience a significant reduction in pain and stiffness, better adherence to treatment regimens, and improved quality of life. Additionally, psychologists and occupational therapists contribute to reducing work absenteeism and social isolation, facilitating patients’ active reintegration into their communities.

From our practical experience, digital health technologies, such as mobile apps and wearable devices, are increasingly being explored for their potential to monitor symptoms, track physical activity, and enhance patient engagement. These tools could be integrated into interdisciplinary strategies to provide personalised feedback and improve adherence to treatment plans.

Environmental and lifestyle modifications, including ergonomic adjustments in the workplace, home adaptations, and stress reduction strategies, are also underexplored in current interdisciplinary rheumatoid arthritis research.

Brignon et al. (2020) underline the indispensable role of social support, facilitated through support groups for patients and their families, which reduce feelings of isolation [92]. MacIver et al. (2021) stress that patient education plays a central role in improving treatment adherence and outcomes by providing valuable information about the disease and self-management strategies [93].

Patient-centred approaches, such as patient-led care models, are an emerging area of interest. Few studies have examined how empowering patients to take an active role in interdisciplinary care impacts outcomes, adherence, and satisfaction. Additionally, educational interventions designed with interactive or multimedia strategies could enhance patient engagement and self-management skills.

For advanced stages of rheumatoid arthritis, the interdisciplinary approach becomes even more critical. Combining advanced technologies, such as joint replacement surgery, with intensive physiotherapy, psychological support, and nutritional management significantly enhances patient outcomes.

Innovative tools like real-time biomarker monitoring via wearable biosensors or AI-driven treatment decisions could further personalise care, enabling earlier intervention and optimised therapy adjustments.

The integration of these elements highlights the importance of a cohesive, patient-centred approach to rheumatoid arthritis management. Despite its benefits, interdisciplinary care faces barriers, such as limited access in underserved areas, a lack of standardised protocols, and insufficient consideration of cultural and socioeconomic factors that influence care delivery. Addressing these gaps is crucial for advancing rheumatoid arthritis management.

This comprehensive approach fosters optimal care and supports patients in effectively managing rheumatoid arthritis.

## 4. Discussion

The studies referenced in the discussion section serve to contextualise and interpret the findings by incorporating broader perspectives and complementary insights. These studies explore related concepts, theoretical frameworks, or practical challenges that extend beyond the scope of the results, enabling a deeper understanding of the implications, identifying limitations, and proposing directions for future research.

The findings of this study align with the existing literature, demonstrating the crucial roles of inflammatory markers, imaging methods, and pharmacological therapies in the management of rheumatoid arthritis.

Dervisevic et al. (2024) emphasise that inflammatory markers, such as C-reactive protein (CRP) and erythrocyte sedimentation rate are central to assessing disease activity and systemic inflammation [94]. Similarly, Cierpiak et al. (2024) highlight that CRP is a sensitive and rapid indicator of acute inflammation, reflecting therapeutic response changes [95]. Additionally, Li et al. (2024) argue that haematological ratios (e.g., neutrophil-to-lymphocyte ratio) strongly correlate with disease activity parameters, offering integrated monitoring insights [96]. da Silva et al. (2024) note that elevated CRP levels and low albumin are critical markers indicating high inflammatory activity and the need for treatment adjustments [97].

Thiele, R.G. (2024) underscore the indispensability of imaging techniques for tracking rheumatoid arthritis progression [98]. While conventional radiographs remain valuable for monitoring bone erosions, advanced methods, such as ultrasound and MRI, can detect active inflammation and early tissue changes, even when clinical symptoms are under control.

The role of non-steroidal anti-inflammatory drugs (NSAIDs) is well-documented in the literature. Shukla et al. (2024) assert that NSAIDs are essential for reducing joint pain and stiffness, providing rapid symptomatic relief from inflammation [99]. However, Hua et al. (2020) caution that long-term use may be associated with gastrointestinal and cardiovascular risks, particularly in patients with comorbidities [100].

Corticosteroids also remain an important class of drugs in rheumatoid arthritis management. Chang et al. (2024) emphasise their efficacy in rapidly controlling severe inflammation during acute phases but note that long-term use is limited due to adverse effects, such as osteoporosis and steroid-induced diabetes [101].

As highlighted in the study by Pavlov-Dolijanovic et al. (2023), elderly-onset rheumatoid arthritis (EORA) presents unique diagnostic and prognostic challenges compared to rheumatoid arthritis in younger populations [102]. The authors emphasize that EORA often manifests with atypical symptoms, such as polymyalgia-like presentations, making early and accurate diagnosis more complex. Furthermore, coexisting comorbidities in elderly patients frequently mask rheumatoid arthritis symptoms, potentially delaying the initiation of appropriate therapies. This underscores the critical need for interdisciplinary approaches in rheumatoid arthritis management, where specialists in geriatrics, rheumatology, and diagnostic imaging collaborate to improve diagnostic accuracy and patient outcomes. These findings align with the broader theme of integrating multiple disciplines to tackle complex disease presentations.

Singh et al. (2024) contribute to this discussion by exploring the role of nanotechnological advancements in disease-modifying antirheumatic drugs (DMARDs) [103]. Their review highlights the potential of nanotechnology to enhance the delivery, efficacy, and safety profiles of DMARDs, particularly in overcoming challenges associated with drug resistance and systemic side effects. From a diagnostic perspective, nanotechnology-based biosensors and biochips hold promise for the real-time monitoring of biomarkers associated with rheumatoid arthritis progression and treatment response. These innovations reinforce the need for integrating cutting-edge technologies into rheumatoid arthritis diagnostic and prognostic workflows to ensure more personalized and precise management strategies.

Inchingolo et al. (2024) provide a broader perspective on the management of rheumatoid arthritis in primary care, emphasizing the importance of early diagnosis and multidisciplinary care [104]. The authors advocate for better integration of diagnostic tools, such as imaging and molecular diagnostics, into primary care settings to facilitate early detection and intervention. This is particularly relevant in addressing barriers, such as limited access to specialist care in resource-constrained environments. Their findings resonate with our analysis, which highlights the logistical and infrastructural barriers to implementing advanced diagnostic tools and artificial intelligence (AI) technologies in healthcare systems.

The study by Andronache et al. (2023) adds an important dimension by discussing the intersection of rheumatoid arthritis management with comorbidities, such as interstitial lung disease (ILD) [105]. Their review emphasizes the need for diagnostic precision in identifying early signs of ILD in rheumatoid arthritis patients, as well as the careful selection of therapies to minimize pulmonary complications. This is consistent with the role of advanced diagnostic imaging and biomarkers in detecting subclinical disease manifestations, which are essential for tailoring treatment plans to individual patient needs.

Bedeković et al. (2023) highlight the role of inflammatory cytokines in the pathogenesis of rheumatoid arthritis and their contribution to secondary complications, such as atherosclerosis [106]. Their findings support the integration of molecular diagnostics and biomarker analysis into rheumatoid arthritis management to monitor inflammatory activity and cardiovascular risk. This aligns with our findings, which underscore the growing importance of integrating prognostic tools into rheumatoid arthritis care to address both disease-specific and systemic complications.

The integration of inflammatory markers, imaging methods, and pharmacological therapies facilitates personalised treatment, enabling optimal disease control and the prevention of long-term disabilities. Studies highlight the necessity of a multidisciplinary approach based on careful monitoring and prompt intervention to minimise the risks associated with rheumatoid arthritis progression.

Despite the comprehensive management strategies outlined, several gaps and challenges remain in the treatment and monitoring of rheumatoid arthritis.

In the role of NSAIDs, while they are effective for symptomatic relief, their inability to alter disease progression and the risks associated with long-term use, such as gastrointestinal and cardiovascular complications, highlight the need for safer, more sustainable alternatives. The lack of robust strategies to personalise NSAID use based on patient-specific risk profiles is also a concern.

Corticosteroids provide rapid control of inflammation, but their significant side effects, particularly with prolonged use, limit their utility. There is insufficient evidence on optimal dosing regimens that minimise risks while maximising benefits. Furthermore, the long-term impacts of corticosteroid use in combination with other rheumatoid arthritis treatments remain underexplored.

For DMARDs, conventional options like methotrexate are accessible and effective but have a slower onset of action and potential side effects requiring frequent monitoring. Biologic DMARDs, while highly effective for severe or refractory cases, are associated with high costs and a risk of infections, including the reactivation of latent diseases. Research into cost-effective biologics or biomarkers to predict treatment response remains limited.

In terms of treatment monitoring, while inflammatory markers like CRP and ESR are essential, their lack of specificity and potential variability due to non-RA factors can lead to challenges in accurately assessing disease activity. Advanced imaging methods, like MRI and ultrasound, provide greater precision but are not standardised across clinical settings, and their accessibility and cost may limit their routine use.

Interdisciplinary approaches, though recognised as essential, face practical barriers in implementation, such as poor coordination between specialists and insufficient patient access to multidisciplinary teams. Additionally, non-pharmacological interventions, such as dietary strategies and psychosocial support, lack standardised protocols and strong evidence from clinical trials to substantiate their integration into routine care.

The updated EULAR recommendations regarding the management of rheumatoid arthritis highlight the importance of early intervention, with the aim of achieving remission or low disease activity. Treatment should be initiated as soon as possible after the diagnosis is confirmed—preferably within the first three months from the onset of symptoms—as the early therapeutic window offers the best chance of controlling the disease and preventing irreversible joint damage [63].

The first therapeutic step recommended by EULAR involves the administration of a conventional synthetic DMARD (csDMARD), typically methotrexate, with a progressively escalated dosage [62]. This is most often combined with low-dose glucocorticoids, administered for short periods, to achieve the rapid control of inflammation [52]. In cases of contraindication or intolerance to methotrexate, leflunomide, or sulfasalazine may be used as alternative options [60].

If, after 3 to 6 months of treatment with an optimally dosed csDMARD, the therapeutic target (remission or low disease activity) is not achieved, treatment should be intensified by adding a biological DMARD (bDMARD) or a JAK inhibitor (tsDMARD) [65]. The choice between these classes should be based on the patient’s profile, comorbidities, and preferences, taking into account the efficacy and safety demonstrated in clinical trials [63]. EULAR particularly recommends the use of TNFα inhibitors (such as etanercept, adalimumab, infliximab, certolizumab pegol, and golimumab) as the first-line biological therapy; although, other mechanisms of action (e.g., abatacept, tocilizumab, rituximab) may also be considered [59].

Regular disease activity assessment is essential to guide treatment decisions, necessitating frequent monitoring using validated scoring systems. The DAS28 (Disease Activity Score in 28 joints) is among the most commonly used clinical tools, incorporating clinical examination data (tender and swollen joint counts), a biological marker of inflammation (ESR or CRP), and the patient’s global assessment. A DAS28 score below 2.6 defines remission, while a score between 2.6 and 3.2 indicates low disease activity. Other widely used scores include the SDAI (Simplified Disease Activity Index) and the CDAI (Clinical Disease Activity Index), which are based on clinical evaluations, with SDAI also including CRP [63].

EULAR recommends frequent monitoring, typically at 1–3-month intervals during active phases of the disease, with appropriate treatment adjustments if the therapeutic target is not met. Once remission is achieved and maintained, the gradual tapering of medication may be considered. However, complete discontinuation of all therapies, particularly biologics, should generally be avoided to prevent disease flares [53].

This structured and adaptive treatment approach aligns with the “treat-to-target” principle, which focuses on reaching a clearly defined therapeutic goal through progressive treatment modifications, tailored to the individual patient’s response. Integrating these principles into clinical practice requires ongoing interdisciplinary collaboration between rheumatologists, general practitioners, rehabilitation specialists, and, when appropriate, psychologists or nutritionists, in order to provide comprehensive, patient-centred care [50].

Modern rehabilitation technologies are assuming an increasingly significant role in the comprehensive management of rheumatoid arthritis, offering tailored solutions aimed at reducing pain, maintaining functionality, and preventing the progression of joint deformities [86]. Rehabilitation now goes beyond traditional physical exercises, integrating advanced technologies that enable a more efficient and personalised recovery process.

In clinical practice, intelligent dynamic orthoses are employed, which adapt to the patient’s biomechanical needs through sensors that provide real-time feedback. These devices contribute to joint stabilisation and reduce stress on inflamed structures, thereby helping to prevent the worsening of lesions. In specialised rehabilitation centres, exoskeletons and robotic equipment have been introduced to assist movement in both the upper and lower limbs, facilitating rehabilitation exercises even in severe cases of joint impairment. This technology allows for the controlled and safe repetition of movements, supporting the restoration of coordination and muscle strength [82].

Virtual reality (VR) is also being used increasingly for therapeutic purposes, creating interactive and stimulating environments in which patients are encouraged to actively engage in exercises, overcoming motivational or psychological barriers. Recent studies indicate increased treatment adherence and a notable improvement in joint mobility when VR platforms are tailored to the specific needs of patients with rheumatoid arthritis [88].

Another innovative component is telemedicine, which enables the remote monitoring and guidance of patients. Through mobile applications or web platforms, therapists can track patient progress, adjust exercises according to individual responses, and maintain ongoing communication—an essential factor for ensuring continuity of the rehabilitation process [84].

Furthermore, complementary therapies, such as functional electrical stimulation (FES), transcutaneous electrical nerve stimulation (TENS), or low-intensity shockwave therapy, are frequently incorporated into personalised treatment plans, demonstrating beneficial effects in reducing pain and improving joint function [86,90].

In addition to the motor component, nutrition plays a significant role in the progression of rheumatoid arthritis. Recent research has highlighted strong correlations between dietary patterns, the levels of systemic inflammation, and the severity of clinical symptoms [87]. Although rheumatoid arthritis is an autoimmune condition, rather than one directly caused by diet, certain foods and dietary habits can either exacerbate or alleviate disease manifestations.

A diet rich in anti-inflammatory compounds helps reduce disease activity by lowering the levels of pro-inflammatory cytokines, such as interleukin-6 and TNF-alpha, and by supporting a balanced gut microbiome, which plays a vital role in modulating the immune response [87].

Protective foods include, first and foremost, oily fish, such as salmon, sardines, or mackerel, which are valuable sources of omega-3 fatty acids. These fatty acids reduce systemic inflammation and are associated with improvements in joint symptoms, including reductions in morning stiffness and pain. Fresh fruits and vegetables, particularly those rich in antioxidants, help to neutralise oxidative stress, a mechanism implicated in joint destruction.

Moreover, whole grains, legumes, and extra virgin olive oil have shown benefits in reducing inflammation and maintaining optimal body weight, a key factor in disease control. Natural yoghurt, kefir, and other fermented foods support a healthy gut flora, with a direct impact on the immune system [87].

On the other hand, certain food categories are considered potentially harmful for rheumatoid arthritis patients. Trans fats, excessive saturated fats, and ultra-processed foods promote chronic inflammation. The frequent consumption of processed red meat, refined sugar, sweetened beverages, and pastries is associated with higher disease activity and a faster decline in general health. Additionally, the high intake of salt and alcohol may interfere with pharmacological treatments and worsen fluid retention, thereby exacerbating joint symptoms [87].

A balanced diet, tailored to the patient’s needs, can become a powerful ally in the therapeutic strategy for rheumatoid arthritis. Collaboration with a nutritionist is essential for developing a personalised dietary plan that takes into account existing comorbidities, cultural preferences, and the patient’s capacity to adhere to long-term recommendations. Therefore, nutritional intervention should not be seen in isolation but rather as an integral part of an interdisciplinary approach, in which each component contributes to disease stabilisation and the improvement of quality of life [84,85].

## 5. Future Perspectives in the Management of Rheumatoid Arthritis

As the understanding of rheumatoid arthritis continues to evolve, future strategies in its management are increasingly focusing on precision medicine, technological integration, and holistic care models. The intersection of advanced rehabilitation technologies, digital health, and individualised nutritional interventions offers promising pathways for improving disease outcomes and enhancing patients’ quality of life.

### 5.1. Expansion of Digital Rehabilitation Platforms

In the coming years, we anticipate a significant expansion in the use of digital rehabilitation platforms incorporating AI-driven algorithms and machine learning to deliver real-time, adaptive therapy plans. These systems will be capable of analysing movement patterns, pain responses, and patient engagement levels to automatically adjust rehabilitation protocols in accordance with individual progress. The integration of wearable sensors and smart textiles will further enhance the accuracy of remote monitoring, allowing clinicians to collect detailed biomechanical data outside clinical settings and to intervene promptly when deviations from the recovery trajectory are detected.

Additionally, robot-assisted rehabilitation is expected to become more accessible and refined. Future generations of lightweight, modular exoskeletons could provide support not only during clinical sessions but also in daily life, contributing to the maintenance of independence and joint protection in patients with advanced disease.

### 5.2. Virtual and Augmented Reality in Personalised Therapy

While virtual reality (VR) is already demonstrating therapeutic potential, augmented reality (AR) and mixed reality (MR) environments are likely to revolutionise the rehabilitation landscape by blending digital content with the physical world. These technologies will support the development of gamified, motivational platforms, fostering greater adherence and engagement through interactive, personalised experiences. Importantly, they may offer significant benefits in addressing psychosocial components of chronic disease, such as depression, anxiety, and fatigue, through immersive cognitive behavioural therapy modules integrated into physical therapy routines.

### 5.3. The Role of Artificial Intelligence in Clinical Decision Making

Artificial intelligence (AI) and big data analytics are expected to play an increasingly central role in the stratification of patients, prediction of flare-ups, and optimisation of treatment pathways. Predictive models drawing on multi-dimensional data—from genomics and proteomics to lifestyle factors—will help identify which patients are more likely to respond to specific biologics, DMARDs, or non-pharmacological interventions. This will facilitate the design of truly personalised treatment regimens, improving long-term outcomes and reducing unnecessary medication exposure.

### 5.4. Integration of Personalised Nutrition into Routine Care

In the future, nutrition will likely become a core component of rheumatoid arthritis management, rather than an adjunctive consideration. Advances in nutritional genomics and metabolomics will enable clinicians to tailor dietary interventions based on each patient’s genetic profile, gut microbiota composition, and metabolic state. Such approaches may optimise anti-inflammatory responses, minimise drug–nutrient interactions, and enhance the efficacy of conventional therapies.

Moreover, AI-powered dietary monitoring tools, including smartphone apps with image recognition and nutrient tracking capabilities, will support real-time dietary assessment and adherence, ensuring greater patient autonomy and clinician insight. Combined with behavioural coaching modules and tele-nutrition services, these tools will promote sustainable dietary changes, particularly in individuals with low health literacy or complex comorbidities.

### 5.5. Towards an Interdisciplinary, Patient-Centred Model

Future care pathways will increasingly embrace a multidisciplinary, patient-centred framework, in which rheumatologists, physiotherapists, rehabilitation engineers, psychologists, nutritionists, and digital health specialists collaborate seamlessly. Interoperable digital health records, shared treatment dashboards, and patient-reported outcome tracking will support a coordinated, responsive system of care delivery.

Importantly, the success of these innovations will depend on addressing accessibility, digital equity, and training needs, particularly among older populations and in under-resourced settings. Investment in patient education, digital infrastructure, and clinician digital literacy will be crucial in ensuring that technological and nutritional advances benefit all individuals living with rheumatoid arthritis, not only those in high-income urban centres.

## 6. Conclusions

The future of rheumatoid arthritis management lies in personalisation, integration, and innovation. By leveraging the synergistic potential of modern rehabilitation technologies, personalised dietary strategies, and data-driven care models, healthcare systems can move towards a proactive, adaptive, and patient-empowered paradigm. These developments promise not only improved clinical outcomes but also a transformative impact on the lived experience of rheumatoid arthritis, supporting autonomy, resilience, and well-being throughout the disease journey.

The key findings of this review emphasise the importance of an interdisciplinary and personalised approach in the treatment and management of rheumatoid arthritis. The integration of diverse medical specialities and the combination of pharmacological and non-pharmacological therapies enable more effective disease control, slower progression, and improved quality of life for patients.

Close monitoring of treatment response, through the evaluation of inflammatory markers and the use of advanced imaging techniques, is essential for therapy adjustments and the prevention of complications. Psychosocial support, patient education, and physical rehabilitation are vital components of successful management, providing patients with the tools needed for better self-management of their condition.

The prognosis in rheumatoid arthritis is a key aspect of disease management, directly influencing patients’ quality of life and treatment outcomes. Early and accurate diagnosis plays a crucial role in improving prognosis, particularly in cases complicated by comorbidities or late-onset forms of the disease. The timely identification of clinical and subclinical signs allows for the rapid initiation of treatment and reduces the risk of functional deterioration.

Emerging technologies, such as nanotechnology, biosensors, and molecular diagnostic tools, bring significant benefits in monitoring disease progression and personalising treatment. These methods enable precise tracking of specific biomarkers that indicate disease progression or therapeutic response. By implementing these advances, patients’ long-term prognosis can be optimised through prompt adjustments to therapeutic interventions and by minimising the risks associated with systemic complications.

Monitoring the pulmonary and cardiovascular manifestations associated with rheumatoid arthritis is essential to preventing severe complications. Interdisciplinary approaches, involving advanced imaging and diagnostic technologies, support the early identification of these risks, contributing to better long-term outcomes. Additionally, the continuous analysis of inflammatory factors and associated comorbidities provides a more comprehensive understanding of disease progression and its risks.

The integration of technological advances into clinical practice, combined with an individualised approach to disease management, can significantly enhance the prognosis for patients with rheumatoid arthritis. Continuous evaluation, tailored therapies, and interdisciplinary collaboration remain central elements in improving the lives of these patients and reducing the burden of the disease on healthcare systems.

Future research should focus on therapeutic innovations and the identification of solutions that reduce the burden of rheumatoid arthritis for both patients and healthcare systems.

## Figures and Tables

**Table 1:** The role of NSAIDs in the management of rheumatoid arthritis.

Aspect	Details	References
Primary Objective	Control inflammation, prevent joint destruction, and maintain patient functionality.	[40]
Role of NSAIDs	Reduce pain and joint stiffness by inhibiting COX-1 and COX-2 enzymes, decreasing the production of prostaglandins involved in inflammation and pain.	[41]
Clinical Use	Frequently used in the early stages of the disease or during flare-ups. Recommended as symptomatic treatment without impact on disease progression.	[42]
Choice of NSAID	Depends on the patient’s safety profile, medical history, and individual response. COX-2 selective inhibitors (e.g., celecoxib) pose fewer gastrointestinal risks.	[43]
Common Side Effects	Gastric ulcers, renal insufficiency, and cardiovascular risks, particularly with long-term use.	[44]
Monitoring Required	Careful monitoring to prevent complications and integration into a broader therapeutic plan, including DMARDs.	[45]
Limitations	Do not influence long-term disease progression, being used solely for symptomatic relief.	[46]
Benefits	Rapid control of inflammatory symptoms and improved quality of life during critical phases of the disease.	[47]
Importance in Treatment	Part of an integrated therapeutic plan, used judiciously to balance benefits and risks.	[48]

COX-1: cyclooxygenase-1, COX-2: cyclooxygenase-2.

**Table 2:** The role of corticosteroids in the management of rheumatoid arthritis.

Aspect	Details	References
Mechanism of Action	Mimic the action of glucocorticoids produced by the adrenal glands, exerting anti-inflammatory and immunosuppressive effects. Inhibit the production of inflammatory cytokines and reduce immune cell activity.	[49]
Clinical Use	Rapidly control inflammation and alleviate acute symptoms, particularly useful during disease flares or as “bridging therapy” before the effects of DMARDs take hold.	[24]
Benefits	Quickly reduce pain, swelling, and joint stiffness. Slow joint damage progression in early stages and help maintain inflammation control in severe forms.	[50]
Administration	Low doses for long-term inflammation control or higher doses for short-term intervention during acute crises.	[51]
Adverse Effects	Osteoporosis, increased blood pressure, steroid-induced diabetes, weight gain, higher infection risk, Cushing’s syndrome, skin fragility, and muscle weakness.	[52]
Monitoring Required	Dose adjustments to the minimum necessary. Monitor side effects and prevent osteoporosis using calcium, vitamin D supplementation, or anti-resorptive therapy.	[53]
Limitations	Significant adverse effects limit long-term use. Requires careful management to minimise risks.	[54]
Role in Treatment	A valuable tool for rapid inflammation control, particularly in severe cases, but should be used responsibly as part of a comprehensive therapeutic plan.	[55]

DMARDs: disease-modifying antirheumatic drugs.

**Table 3:** Comparison between conventional and biologic DMARDs in the treatment of rheumatoid arthritis.

Aspect	Conventional DMARDs	Biologic DMARDs	References
Definition	Conventional drugs that modulate the immune response to reduce inflammation and slow disease progression.	Biologic agents that specifically target molecules or cells involved in the inflammatory immune response.	[56,57]
Examples	Methotrexate, Sulfasalazine, Hydroxychloroquine, Leflunomide.	Infliximab, Adalimumab, Tocilizumab, Etanercept.	[58,59]
Mechanism of Action	Inhibit immune cell proliferation and reduce the synthesis of inflammatory cytokines.	Block specific immune targets, such as TNF-α, interleukins, or other inflammatory proteins.	[60,61]
Efficacy	Gold standard for rheumatoid arthritis treatment (e.g., Methotrexate). Can be used as monotherapy or in combination with other drugs.	Highly effective, particularly for patients unresponsive to conventional DMARDs.	[62,63]
Route of Administration	Usually, oral.	Typically, injectable or via infusion. Recently, some synthetic biologic therapies (e.g., JAK inhibitors) are available orally.	[64,65]
Time to Effect	Requires weeks or months to achieve maximum effect.	Faster clinical response compared to conventional DMARDs.	[57,66]
Risks and Side Effects	Hepatotoxicity, bone marrow suppression, and allergic reactions. Frequent monitoring of liver function and blood counts required.	Increased risk of severe infections, including latent tuberculosis. Injection site or secondary autoimmune reactions may occur.	[67,68,69]
Costs	Lower costs, accessible in most healthcare systems.	High costs, usually reserved for severe cases or those unresponsive to conventional DMARDs.	[70,71]
Monitoring	Requires liver function tests and blood counts.	Regular evaluations for infections and adverse reactions needed.	[72,73]
Benefits	First-line treatment, effective for most patients.	Second-line treatment with high efficacy in refractory cases.	[74,75]
Limitations	May require combination therapy for optimal response. Slower onset of action.	High costs and increased risk of severe side effects.	[2,76]

## Data Availability

The database used is from the review. Considering data protection regulation, we cannot provide access to the original database.
